# A mapping review and critique of the literature on translation, dissemination, and implementation capacity building initiatives for different audiences

**DOI:** 10.1186/s43058-025-00717-w

**Published:** 2025-04-03

**Authors:** Ana A. Baumann, Danielle R. Adams, Laura-Mae Baldwin, Rachel G. Tabak, Sara Malone, Maura M. Kepper, Anita D. Misra-Hebert, Kathleen R. Stevens, Maria E. Fernandez, Sunil Kripalani

**Affiliations:** 1 Division of Public Health Sciences, Department of Surgery, Washington University School of Medicine, St. Louis, MO USA; 2 School of Social Work, College of Health Sciences, University of Missouri, Columbia, MO USA; 3 Department of Family Medicine, University of Washington, Seattle, WA USA; 4 School of Public Health, Washington University in St. Louis, St. Louis, MO USA; 5 Cleveland Clinic Lerner College of Medicine of Case Western Reserve University School of Medicine, Cleveland, OH USA; 6 University of Texas Health Science Center San Antonio, San Antonio, TX USA; 7 Institute for Implementation Science, University of Texas Health Science Center at Houston, Houston, TX USA; 8 Vanderbilt University Medical Center, Nashville, TN USA

**Keywords:** Translational, Dissemination, Implementation, Capacity building, Competencies

## Abstract

**Background:**

Capacity building is critical for research and practice as the fields of dissemination, implementation and translation science continue to grow. Some scholars state that capacity building should be grounded in competencies. However, the fields are unclear in determining which competencies are relevant for whom, including the content and appropriate level of information and skills for different roles. The goal of this study was to catalogue competencies across current D&I capacity building initiatives.

**Methods:**

We conducted a mapping review to examine to what extent are theories or frameworks used to guide capacity building, who is being trained, to what extent do capacity building initiatives include a health equity focus, which competencies are being outlined or suggested, how are they being defined, and whether the competencies can be organized along different roles of participants. As a mapping review, we broadly searched for papers using the keywords “training D&I” OR “training implementation” OR “training translation” OR “training dissemination” and included debate and empirical papers about capacity building initiatives in the sample.

**Results:**

A total of 42 articles (from 2011 to 2024) were reviewed, including training development and/or evaluation (*n* = 25) and conceptual (*n* = 17) articles. Of the training articles, 13 (52%) specified a framework that guided training. Participants in training included graduate students, researchers, practitioners, and mixed audiences. Fourteen (56%) of the trainings were conducted in the USA, seven (28%) in Canada and other countries. The length of training ranged from two days to two years. Four trainings had an explicit focus on equity. A total of 307 unique competencies were identified and divided into themes: Knowledge, Skills, Engagement with Other Disciplines, Equity, Attitude and Relational Aspects, Capacity Building, Quality Improvement, and Mentorship.

**Conclusions:**

While there are many D&I capacity building initiatives, we found little consistency in competencies that guided training activities for diverse audiences. Few training activities explicitly identified guiding theories or frameworks or tailored competencies toward different levels of interest in D&I research. Even fewer had an explicit focus on health equity. As the fields continue to foster capacity building programs, it will be important to think critically about the types of competencies we are developing for whom, how, and why.

Contributions to the literature

- This study provides a critical step in the dissemination, implementation and translation capacity building literatures by cataloging competencies related to these sciences, and by offering suggestions for capacity building initiatives.

## Background

Translation, dissemination, and implementation sciences are rapidly growing fields. All three aim to (1) move research into practice [1], (2) accelerate the uptake of evidence-based interventions, policies, guidelines, and practices into clinical and community settings [2], and (3) examine factors that affect the spread and uptake of knowledge [2]. These three sciences are related, bidirectional, and have similar goals: to identify practices and principles to improve healthcare delivery and health outcomes [1]. In this paper, we will use the dissemination & implementation (D&I) acronym to encompass these three sciences.

Capacity building is critical for research and practice as these fields continue to grow. Some scholars state that capacity building should be grounded in competencies [3, 4] which provide a framework for teaching learners certain skills [5] through knowledge acquisition and practice [6]. The general assumption of a competency-based approach to capacity building is that an occupation “can be broken into smaller elements of defined knowledge and skills (competencies), and that achievement of an accepted level in each of these domains will lead to overall proficiency” [5]. Competency frameworks also help delineate the degree to which knowledge and skills may differ across these three different sciences [3, 7–10].

Despite advances in capacity building in D&I, gaps remain in outlining various options for building capacity that is most relevant to differing potential groups of trainees. Individuals working in D&I may have different levels, forms of engagement, and experience with the application of fundamental principles. For example, expanding on Tabak et al. [11], we pose at least three phenotypes of people engaged with the D&I fields: 1) the collaborator who is interested in a basic level of knowledge to be able to work effectively with a D&I expert, 2) the scholar who uses D&I science in his/her/their research, and 3) the expert methodologist who seeks to advance the fields of D&I science. In addition to these different levels of research engagement with the science, a person may want to know how to apply the D&I sciences as a practitioner, healthcare leader, public health practitioner, or someone interested in policymaking [12–17] Accordingly, scholars have underscored the need for training practitioners, implementation champions, community and healthcare leaders, policymakers, and administrators [15, 18] to generate a D&I workforce. The goal of such a diverse workforce would be to apply translational knowledge responsive to community needs and accelerate the uptake of evidence-based interventions, policies, and practices [19].

The fields of D&I have recently increased their attention to health equity and to healthcare equity [12, 20], with one of the assumptions being that embedding D&I in healthcare settings centered in equity perspectives could promote equity in healthcare practice and research [15, 21]. We define health equity as not only the absence of obstacles to health and well-being for all populations across multiple sectors and societal contexts, but also the presence of quality of life [22]. We define healthcare equity as the just and appropriate access and utilization of healthcare services without variation due to demographic, economic or political factors [23]. To achieve health equity and healthcare equity, we need to address the historical and current systemic factors that affect healthcare delivery practices and policies. We also need to address the non-medical drivers of care that affect access and utilization [15]. Data has shown, however, that even if researchers and practitioners are motivated to engage in equity-oriented D&I research (i.e., research that explicitly and carefully attends to the culture, history, values, assess and needs of the community [20]), there is a lack of capacity building initiatives in this space [24]. As such, one could argue that capacity building initiatives need to have greater attention to the underlying systemic factors that affect the availability and delivery of quality care and their alignment with cultural perspectives, history, assets, and needs of the community served [12, 25–30] through the development of a representative workforce. This workforce – including providers, healthcare leaders, and policymakers—needs to be able to generate and apply knowledge that is responsive to community needs and accelerates the implementation of evidence-based practices and guidelines while promoting the delivery and monitoring of equitable healthcare [19].

As more individuals are drawn to learning about D&I sciences, we believe it is essential to: (a) determine which competencies are relevant for whom, including the content and appropriate level of information and skills for different roles; (b) understand and address the intersection between D&I and health equity [12, 15, 31]; and (c) provide opportunities for training those most interested in applying D&I science, including teaching practical skill such as facilitation roles and problem-solving skills during capacity-building initiatives [32–34].

We use the term “capacity building” instead of training to reflect the different types of initiatives that may go beyond traditional teaching of D&I competencies. The focus here is on human capacity building and not on resource or infrastructure. While there is no consistent definition on capacity building [35], we conceptualize here the word “capacity” as the ability to carry out a given task, and “building” as the act of developing something. As such, capacity building refers to the development of human competencies to achieve a set goal. The goal of this study was to identify the state of current D&I capacity-building initiatives for expanding dissemination, translation, and implementation research and practice.

Lists of competencies for D&I capacity buildings have been developed using a variety of methods, including card sorting, Delphi methods, concept mapping, and surveys [36]. The challenge with these lists is that they do not necessarily define competencies and do not follow a common lexicon across different initiatives. In fact, since 2020, scholars [8, 36] have asked for standardization of capacity building reporting, including clear outlining of competencies used to train in D&I, to better understand the findings across different contexts and scientific professions. The argument is that, by having clear definitions of competencies using a common lexicon, we can then evaluate which competencies are needed for whom (e.g., practitioners, researchers), at which level (e.g., beginners, advanced) across the different capacity building initiatives. Our study differs from other reviews in that it provides an overarching library of current competencies identified by the authors from different types of capacity building initiatives. It also differs from other reviews in that we are not focused on a specific audience [37, 38]. We do not aim to define further the competencies that we identified; we believe that this is a next step in our work, but rather we present the state of the art of competencies in D&I competencies to increase awareness of the current development of the field and provide recommendations for developers of capacity building initiatives.

## Methods

We conducted a mapping review to explore and map the literature available on this topic, and to identify key concepts and gaps in the D&I capacity building literature [39, 40], and to inform future more structured reviews, such as a scoping review [41]. We chose a mapping review because this type of review is meant to answer more deductive questions. Different from scoping reviews, which aim to systematically identify and map the breadth of evidence available on a particular topic, mapping reviews are meant to catalog the evidence gaps in a broader topic area, collate and describe the available evidence relating to the question of interest. In other words, they yield higher level data extraction and give a broader focus of the field by answering the question “what do we know about a topic,” or “what and where does research exist in a particular area” [42]. Mapping reviews, therefore, are meant to be broad and provide an overview of the field [43].

### Identifying the research question

Our primary research question was: What is the state of the art – or the most recent stages of development- of current D&I capacity building initiatives? Our secondary questions were: a) to what extent are theories or frameworks used to guide capacity building, (b) who is being trained, (c) what is the length of training, (d) to what extent do capacity building initiatives include a health equity focus, (e) which competencies are being outlined or suggested, and how are they being defined, and (f) can the competencies be organized along different roles of participants.

### Identifying and categorizing the articles

AB conducted an initial search in Feb 2021 using GoogleScholar to identify articles related to training in dissemination and implementation using the keywords “training D&I” OR “training implementation” OR “training translation” OR “training dissemination.” The search was updated in June 2022, and newer articles were added based on co-author recommendation until September of 2023. Another search was done in January of 2025 when reviews for the submitted manuscript were received. Because this is a mapping review, we broadly included papers related to capacity building initiatives in D&I, including empirical and conceptual papers. Papers related to other types of trainings (e.g., community engagement) that did not explicitly talk about capacity building initiatives in D&I sciences were not included in the final sample. All papers included in the sample were reviewed in full by both AB and DA.

We categorized articles in two ways: (1) Training development and/or evaluation articles, which were those describing the development of some sort of didactic course (e.g., workshop, training, expert consultation) that was meant to develop capacity of people, papers evaluating training, or papers describing both the development and evaluation trainings, and (2) Conceptual articles, which offered commentaries, perspectives, arguments, or recommendations about D&I capacity building that were not essentially based on empirical research, but were grounded in the literature and expert observations. The conceptual articles also included review articles summarizing capacity-building initiatives.

### Charting the data

During the full-text review, we extracted the following data from each article: the theory or framework used to guide the capacity building initiatives, the participants, the length of the training, whether it had an equity focus, and the competencies outlined in the article. AB and DA charted the data; AB compiled the competencies, and DA charted the other components of the articles. AB and DA met bi-monthly to reconcile discrepancies.

### Collating, summarizing, and reporting the data

Competencies were compiled from all papers (i.e., training development and debates). They were literally copied and pasted from what the authors wrote in the papers, with no interpretation. Because this is a mapping review, and we had a large and varied sample of papers that yielded many competencies. To organize the discrete competencies, AB used a rapid coding of the competencies and sorted them into bins/themes and all authors provided feedback and further recommendations. Further details about the competencies per paper can be available upon request from the corresponding author. The data about competencies per paper are not publicly available due to its potential sensitive comparisons across capacity initiatives, which is not the goal of the present study.

Competencies were sorted into themes: Knowledge, Skills, Equity, Engagement with Other Disciplines, Attitudes and Relational Aspects, Capacity Building/Infrastructure, Quality Improvement and Mentoring. Competencies were then tallied by frequency in each theme.

### Defining the competency themes

The 307 identified competencies were first divided into two large themes: knowledge or skills. A competency was coded in the *knowledge* theme if the verbs used to describe it were “identify”, “define” or “describe.” A competency was coded in the *skill* theme if it referred to “use”, “apply”, “employ” or another similar active verb. The rationale for first defining competencies into knowledge and skills was related to the education, medicine, and management literatures that reminds us that knowledge acquisition does not guarantee the successful application of that same knowledge, and as such it is important to also teach skills on how to apply the knowledge [44].

The competencies that did not fit either knowledge or skills themes because they often did not have the verbs outlined above were thematically coded as “Engagement with other disciplines”, “Equity”, “Attitude and Relational Aspects”, “Capacity Building/Infrastructure”, “Quality Improvement” and “Mentoring.” Note that these are subjective themes.

Competencies coded as “Engagement with other disciplines” involved actions related to either collaborating with other disciplines or articulating how other disciplines could foster D&I. Competencies coded in “Equity” broadly involved competencies that exposed participants to methods aimed at engaging community members in the research and implementation process (e.g., engaging stakeholders in identifying outcomes and measures). While one could separate the “Attitude and Relational Aspects” theme into attitude characteristics (e.g., being honest, positive leader) and relational aspects (e.g., being able to work in teams), we posit that these two could also be related: attitudes are affected by context and relational aspects; similarly, relationship dynamics are affected by people’s attitudes [45, 46]. Therefore, we bundled these two constructs in one theme.

Competencies in the “Capacity Building/Infrastructure” involve the ability to build capacity to implement the project/study such as staff training and acquisition of funding. The competencies in “Quality Improvement” are related to using data for monitoring and improving the implementation process, including the development of logic models and an evaluation process. Finally, competencies in the “Mentoring” category involve either receiving and/or providing mentoring.

To gather feedback on these themes, we presented different versions of these groupings to groups of experts who provided further suggestions, including: poster sessions at the 2021 and 2022 Conferences on the Science of Dissemination and Implementation in Health, through internal presentations at our universities, through presentations to the Clinical and Translational Science Award (CTSA) D&I Working Group, and at meetings with our network of D&I research peers in our universities. During these presentations, and among our internal authorship group, we asked for feedback on whether the themes were descriptive of the list of the competencies, and on the rationale for the paper (i.e., the type of review and the value added of this paper compared to the larger literature). Feedback was received in the form of comments during the poster sessions, and during the question-and-answer sections during the presentations. Overall, we received feedback that the categorization of the competencies was useful, that the method (i.e., a mapping review) and this paper added value to the field of D&I capacity building.

## Results

A total of 42 articles from 2011 to 2024 were reviewed, including training development and/or evaluation (*n* = 25) and conceptual articles (*n* = 17). We focus the results below (frameworks, participants, location & length of trainings, and equity) on only training development and/or evaluation articles, given that our focus was on understanding how trainings are designed. For the competencies section, we include all 42 articles in the result summaries.

### Frameworks guiding the trainings

Out of 42 articles reviewed, eighteen (43%) specified a framework that guided training, shown on Table 1. The frameworks were varied, including knowledge translational frameworks and educational frameworks. No two trainings used the same framework.

**Table 1:** Frameworks that guided the capacity building efforts

Citation	Theory / Framework Used
Straus, S.E., Brouwers, M., Johnson, D. et al. Core competencies in the science and practice of knowledge translation: description of a Canadian strategic training initiative. Implementation Sci 6, 127 (2011). 10.1186/1748-5908-6-127 [47]	The UK MRC Framework for Complex Interventions [48]
Meissner HI, Glasgow RE, Vinson CA, Chambers D, Brownson RC, Green LW, Ammerman AS, Weiner BJ, Mittman B. The U.S. training institute for dissemination and implementation research in health. Implement Sci. 2013;8:12. [49]	Adapted version of Ward’s conceptual framework of translating knowledge [50]
Stevens, K. R. (2013). The impact of evidence-based practice in nursing and the next big ideas. Online journal of issues in nursing, 18(2). [51]	The ACE Star Model of Knowledge Transformation [52]
Urquhart, R., Cornelissen, E., Lal, S., Colquhoun, H., Klein, G., Richmond, S., & Witteman, H. O. (2013). A community of practice for knowledge translation trainees: an innovative approach for learning and collaboration. [53]	Framework developed within the collaborative to evaluate its impact as a community of practice
Proctor, E. K., Landsverk, J., Baumann, A. A., Mittman, B. S., Aarons, G. A., Brownson, R. C., … & Chambers, D. (2013). The implementation research institute: training mental health implementation researchers in the United States. Implementation Science, 8(1), 1–12. [54]	Heuristic framework for implementation research in mental health [55]
Estapé‐Garrastazu, E. S., Noboa‐Ramos, C., De Jesús‐Ojeda, L., De Pedro‐Serbiá, Z., Acosta‐Pérez, E., & Camacho‐Feliciano, D. M. (2014). Clinical and translational research capacity building needs in minority medical and health science Hispanic institutions. Clinical and translational science, 7(5), 406–412. [56]	The clinical and translational research core areas and competencies developed by the National Institutes of Health-Clinical and Translational Sciences Award [57]
Osanjo, G. O., Oyugi, J. O., Kibwage, I. O., Mwanda, W. O., Ngugi, E. N., Otieno, F. C., … & Kiarie, J. N. (2015). Building capacity in implementation science research training at the University of Nairobi. Implementation Science, 11(1), 1–9. [58]	“Competency-based medical education (CBME) and adult-learning principles underpin the curriculum. Other curriculum elements that were added and emphasized include innovative teaching strategies, content related to the Kenyan health care system, inter-professional team training, assessment of learning needs, mentoring, development of research skills, and evaluation of the processes.” [58, 59]
Ullrich, C., Mahler, C., Forstner, J. et al. Teaching implementation science in a new Master of Science Program in Germany: a survey of stakeholder expectations. Implementation Sci 12, 55 (2017). 10.1186/s13012-017-0583-y. [60]	The European Framework for Higher Education [61]
Baldwin, J. A., Williamson, H. J., Eaves, E. R., Levin, B. L., Burton, D. L., & Massey, O. T. (2017). Broadening measures of success: results of a behavioral health translational research training program. Implementation Science, 12(1), 1–11. [62]	The Translational Research Impact Scale [63]
Moore, J. E., Rashid, S., Park, J. S., Khan, S., & Straus, S. E. (2018). Longitudinal evaluation of a course to build core competencies in implementation practice. Implementation science, 13, 1–13. [64]	Knowledge-to-action (KTA) [50]
Ramaswamy, R., Mosnier, J., Reed, K., Powell, B. J., & Schenck, A. P. (2019). Building capacity for Public Health 3.0: introducing implementation science into an MPH curriculum. Implementation Science, 14(1), 1–10. [65]	Adapted a model termed design-focused implementation (Fig. 2) to guide our curriculum development. [66]
Albers, B., Metz, A. & Burke, K. Implementation support practitioners – a proposal for consolidating a diverse evidence base. BMC Health Serv Res 20, 368 (2020). 10.1186/s12913-020-05145-1. [34]	The Interactive Systems Framework [67]
Davis, R., Mittman, B., Boyton, M., Keohane, A., Goulding, L., Sandall, J., … & Sevdalis, N. (2020). Developing implementation research capacity: longitudinal evaluation of the King’s College London Implementation Science Masterclass, 2014–2019. Implementation science communications, 1(1), 1–13. [68]	The content of the ISM was developed through reviewing core competencies in knowledge translation [47], taken from a 2011 Canadian training initiative, as well as a 2012 framework for training healthcare professionals in implementation and dissemination science. [69]
Schultes, MT., Aijaz, M., Klug, J. et al. Competences for implementation science: what trainees need to learn and where they learn it. Adv in Health Sci Educ 26, 19–35 (2021). 10.1007/s10459-020-09969-8. [70]	Educational psychology theoretical framework [71]
Rogal, S. S., Jonassaint, C., Ashcraft, L., Freburger, J., Yakovchenko, V., Kislovskiy, Y., … & Chinman, M. (2022). Getting To Implementation (GTI)-Teach: A seven-step approach for teaching the fundamentals of implementation science. Journal of Clinical and Translational Science, 6(1), e100. [72]	Getting to Implementation [72, 73]
Pérez Jolles, M., Willging, C. E., Stadnick, N. A., Crable, E. L., Lengnick-Hall, R., Hawkins, J., & Aarons, G. A. (2022). Understanding implementation research collaborations from a co-creation lens: recommendations for a path forward. Frontiers in health services, 2, 942,658. [74]	Exploration Preparation Implementation Sustainment (EPIS) Framework [75]
Miyamoto, K., Okamoto, R., Koide, K., & Shimodawa, M. (2024). Effect of web-based training on public health nurses’ program implementation capacity: a randomized controlled trial. BMC nursing, 23(1), 678. [76]	Consolidated Framework for Implementation Research (CFIR) [77]
Stevens, K. R., De La Rosa, E., Ferrer, R. L., Finley, E. P., Flores, B. E., Forgione, D. A., … & Wooten, K. C. (2021). Bootstrapping implementation research training: A successful approach for academic health centers. Journal of Clinical and Translational Science, 5(1), e168. [78]	The program was based on an established national curriculum [79], national consensus on dissemination and implementation (D&I) research competencies [3], and recently published NIH/ NCI OpenAccess training materials [80], offering a standardized frame

### Participants

Twenty unique trainings were identified; see Table 2 for details on number and type of participants for each training, as well as additional details on how trainings recruited and selected participants. The majority of trainings (*n* = 11, 55%) recruited a mix of participants, including some variation of graduate students, researchers, practitioners, public health leaders, policy-makers, decision-makers (including clinicians, healthcare managers, and policy makers), and teaching staff. Five (25%) training programs were only offered to researchers, three (15%) training programs were offered only to students (these were Master’s degrees or Master’s-level courses), and one (5%) training program was offered only to public health nurses.

**Table 2:** Training characteristics, recruitment, and selection

Citation	Number and type of participants	Length of training (Short, Medium, Long)	Format (in person, virtual, hybrid)	Location	How people were recruited and selected
Straus, S.E., Brouwers, M., Johnson, D. et al. Core competencies in the science and practice of knowledge translation: description of a Canadian strategic training initiative. *Implementation Science, 6*, 127 (2011). [47]	Average of 30 attendees at summer institute. Graduate students, researchers and trainees from other fields, decision makers	Medium Stream 1: annual Summer Institute, yearly research meetings, and a research practicum if desired by the trainee Stream 2: One day, in person Stream 3: Not listed	In person	Canada	N/A
Meissner HI, Glasgow RE, Vinson CA, Chambers D, Brownson RC, Green LW, Ammerman AS, Weiner BJ, Mittman B. The U.S. training institute for dissemination and implementation research in health. *Implementation Science.* 2013;8:12. [49]	35 doctoral-level health sciences researchers	Short 5-days	In person	United States	**Recruited:** TIDIRH’s call for applications was disseminated through the Office of Behavioral and Social Sciences Research and other NIH listservs containing potentially interested subscribers and announced at the 2011 NIH Conference on the Science of D&I **Selection:** Hold a doctoral level degree, have demonstrated experience and expertise in health science, have a D&I research concept to bring to the institute and develop throughout the training
Urquhart, R., Cornelissen, E., Lal, S., Colquhoun, H., Klein, G., Richmond, S., & Witteman, H. O. (2013). A community of practice for knowledge translation trainees: an innovative approach for learning and collaboration. *Journal of Continuing Education in the Health Professions, 33*(4), 274–281. [53]	123 researchers	Long – Ongoing collaborative	Hybrid	Canada	N/A
Proctor, E. K., Landsverk, J., Baumann, A. A., Mittman, B. S., Aarons, G. A., Brownson, R. C., … & Chambers, D. (2013). The implementation research institute: Training mental health implementation researchers in the United States. *Implementation Science, 8*(1), 1–12. [54]	10 doctoral-level researchers are selected as fellows each year	Long – 2-year fellowship	Hybrid (2 weeks in person, virtual meetings throughout the year	United States	**Recruitment:** Information about the IRI and application process is widely disseminated through relevant email distribution lists, listservs, word-of-mouth, and conference announcements **Selection:** prior or concurrent experience relevant to IR such as intervention development and/or testing, mental health services research, or study of organizational factors in mental health service delivery; experience writing an NIH, VA, or other federal grant; a strong local mentor in the applicant’s home institution who is supportive of the fellow’s grant writing and scholarly publication (the local mentor need not be an expert in IR, but must have a strong record of NIH or VA funding); and access to a clinic/service setting willing to serve as a pilot site for the fellow’s implementation research
Osanjo, G. O., Oyugi, J. O., Kibwage, I. O., Mwanda, W. O., Ngugi, E. N., Otieno, F. C., … & Kiarie, J. N. (2015). Building capacity in implementation science research training at the University of Nairobi. *Implementation Science*, *11*(1), 1–9. [58]	5 master’s level health sciences professionals each year	Long – 2-years total, including 3-months period where fellow would go to a US University for didactic training	Not listed	Kenya	**Recruitment:** Hospitals and health care settings across Kenya were recruited to participate in the program **Selection:** The selection process was framed to achieve a balanced mix of fellows based on educational background, geographical place of work, and gender. Trainees were to be selected from UON, KNH, and MOH health facilities. The minimum qualification for entry was a Master’s degree. Applicants were required to demonstrate motivation to pursue a career in implementation science, be able to attend training for 2 years if accepted, and provide a letter of support from their organization or institution. Preference was given to early-career applicants and those without established major research funding
Ullrich, C., Mahler, C., Forstner, J. et al. Teaching implementation science in a new Master of Science Program in Germany: a survey of stakeholder expectations. *Implementation Science, 12*, 55 (2017). [60]	About 20 bachelor’s-level health sciences students can be accepted each year	Long – 2-year full-time Master of Science program in Health Services Research and IS	Not listed	Germany	**Selection:** Applicants must hold a bachelor degree related to health sciences or health professions and proof of at least basic knowledge in empirical research methods. Students are selected in a two-step process based on grades, practical experience, a motivation letter, and personal interviews
Baldwin, J. A., Williamson, H. J., Eaves, E. R., Levin, B. L., Burton, D. L., & Massey, O. T. (2017). Broadening measures of success: results of a behavioral health translational research training program. *Implementation Science,* 12(1), 1–11. [62]	12–15 per cohort. Graduate-level (Master’s and doctoral) researchers and behavioral health practitioners	Long – The graduate certificate program (15 credits) requires four continuous semesters, a total of 18 months, to complete	Hybrid – “Scholars complete online coursework, while also completing their in-person service-learning research project.”	United States	**Recruitment:** Institute scholars were recruited from health- and social services-related graduate academic disciplines, programs **Selection:** Interested applicants applied to the Institute by submitting a personal statement, resume, two letters of recommendation, and official transcripts. Members of the Executive Committee then reviewed the applicants collectively and made final decisions regarding admission to the program
Padek, M., Mir, N., Jacob, R.R. et al. Training scholars in dissemination and implementation research for cancer prevention and control: a mentored approach. *Implementation Science, 13*, 18 (2018). [81]	12 doctoral-level early to mid-career, cancer control researchers	Long – 2-year fellowship	Hybrid- Fellows attended a 5-day summer institute at Washington University in St. Louis to receive didactic, group, and individual instruction on their research area of interest as it pertains to D&I science. Ongoing mentoring relationships occurred over the 2 years of the program, complemented by intermittent webinar sessions with topics chosen by the fellows themselves	United States	**Recruitment:** Emails were sent out through various listservs, flyers were made available at conferences, networking **Selection:** Applicants must have a full-time appointment in a US or international based research setting and a research focus on cancer control. Faculty scored each application based on eight questions regarding overall quality, demonstrated commitment to D&I science, demonstration of experience working in transdisciplinary networks, evidence of research support and potential, likelihood for career development, appropriate methods in concept paper, appropriate topic in concept paper, and potential impact of the work proposed
Moore, J. E., Rashid, S., Park, J. S., Khan, S., & Straus, S. E. (2018). Longitudinal evaluation of a course to build core competencies in implementation practice. *Implementation Science, 13*, 1–13. [64]	17 participants enrolled in the PKT course. Participants included implementation researchers, healthcare professionals, project and grant collaborators, participants of previous KT training initiatives	Medium – 6 months program including 3-day in person workshop	Hybrid – Delivered over 6 months and included a 3-day in-person workshop and 11 synchronous webinars. Instructors used interactive, large-group lectures to present KT theories, models, frameworks, and how to apply these in practice	Canada	**Recruitment:** The PKT course was advertised between using recruitment emails shared with the course developers’ circle of contacts. Recruitment ads were posted in online forums and newsletters **Selection:** Application describing their roles, previous experience with implementation, and their interest in participating in PKT. They were also asked to describe project(s) they worked on, their learning goals, and anticipated benefits of participating in PKT. Two course developers reviewed the 19 applications received to assess alignment of the course objectives with applicants’ learning goals and interest in participating, scope and relevance of the identified project(s), and applicants’ position to impact healthcare outcomes
Ramaswamy, R., Mosnier, J., Reed, K., Powell, B. J., & Schenck, A. P. (2019). Building capacity for Public Health 3.0: introducing implementation science into an MPH curriculum. *Implementation Science*, *14*(1), 1–10. [65]	Total enrollment of 142, of whom 127 have been master’s-level students in the school of public health. The primary target audience for these courses was a cohort of MPH students enrolled in a newly created online MPH program	Medium – semester long courses	Online –Semester long courses were all delivered online with a mix of asynchronous and synchronous content	United States	Students were recruited from existing Master’s in Public Health program at the University of North Carolina
Shete, P. B., Gonzales, R., Ackerman, S., Cattamanchi, A., & Handley, M. A. (2020). The University of California San Francisco (UCSF) training program in implementation science: program experiences and outcomes. *Frontiers in Public Health, 8*, 94. [82]	Between 2008 and 2015, 71 students completed the in person certificate program. Between 2016–2017, 13 students completed the online Certificate Program	Long—Six, 10 week (equivalent to an academic quarter) long courses	In person and Virtual—The original training program was delivered in an in-person format. In 2016, we introduced an online format for the 6 courses	United States	N/A
Davis, R., Mittman, B., Boyton, M., Keohane, A., Goulding, L., Sandall, J., … & Sevdalis, N. (2020). Developing implementation research capacity: longitudinal evaluation of the King’s College London Implementation Science Masterclass, 2014–2019. *Implementation Science Communications, 1*(1), 1–13. [68]	501 delegates have attended the Implementation Science Masterclass. Open to all individuals interested in the application of IS, irrespective of their professional background, where they fall on the career trajectory, or their expertise	Short – 2-days	In person, balance of didactic lectures and interactive workshops	United Kingdom	N/A
Black, A. T., Steinberg, M., Chisholm, A. E., Coldwell, K., Hoens, A. M., Koh, J. C., … & Snow, M. E. (2021). Building capacity for implementation—the KT Challenge. *Implementation Science Communications, 2*, 1–7. [83]	To date, 24 teams have been funded across 4 cohorts, comprising 185 health care professionals (HCPs). Participants have included a wide range of HCPs involving 23 types of practitioners working within a range of practice settings	Long – 2-years of funding to accomplish practice change	Hybrid, two half-day in person workshops paired with online resources, mentorship, and funding	Canada	**Recruitment:** The KT Challenge program was implemented at two health organizations in British Columbia, Canada, and offered to all HCPs employed at the organizations **Selection:** In the letter of interest (LOI), teams identify the practice change they want to implement, document the need for this change in their practice context, and summarize evidence of its effectiveness. The LOIs are formatively reviewed and, in keeping with the capacity building approach of this program, revisions are suggested when required
Friedman, D. B., Escoffery, C., Noblet, S. B., Agnone, C. M., & Flicker, K. J. (2021). Building capacity in implementation science for cancer prevention and control through a research network scholars program. *Journal of Cancer Education*, 1–10. [84]	20 individuals were selected for year 1 of the program. Participants included students (undergraduate through doctoral), postdoctoral fellows, junior faculty, practitioners, and health professionals in the United States	Medium – 9-months	Hybrid—The program is self-paced with some synchronous meetings, including a kickoff meeting, planned webinars with all scholars, and a closing meeting. Scholars work on their projects and either curriculum—NCI’s D&I modules or CPCRN’s PPHEA program. They also are invited to collaborate with a CPCRN workgroup, attend the annual CPCRN meeting	United States	**Recruitment:** An email call for applications which was sent to members of the the Cancer Prevention and Control Research Network (CPCRN) Steering Committee with a request to distribute the email and link to the electronic application form. Steering Committee members distributed the email via student listservs at their home institutions **Selection:** Workgroup project co-directors assigned two reviewers to each application. The review form was guided by the scholar application questions and rated applications on the following criteria using 1 = excellent, 2 = fair, and 3 = poor. Applicants were rated on the following: Evidence of interest in cancer prevention and D&I, clear description of and feasibility of proposed project, goals, and activities, how proposed goals and activities contributed to diversity of the training program, proposed goals, activities, and project fit with CPCRN workgroup and efforts
Vroom, E. B., Albizu-Jacob, A., & Massey, O. T. (2021). Evaluating an implementation science training program: impact on professional research and practice. *Global Implementation Research & Applications*, *1*, 147–159. [85]	Over 90 students have been trained since 2013. Scholars include undergraduate and graduate students from various disciplines as well as current researchers and professionals (i.e., non-degree seeking individuals) working in community settings. Participants include scholars from multiple health and social science disciplines	Long—The program is completed over four consecutive semesters (2-years) and requires scholars to complete 15 credits hours: three online courses (three credits per course) and three service-learning courses (two credits per course)	Hybrid – the Institute for Translational Research Education in Adolescence Drug Abuse (ITRE) requires three online courses and three service-learning courses with in-person seminars and workshops	United States	N/A
Rogal, S. S., Jonassaint, C., Ashcraft, L., Freburger, J., Yakovchenko, V., Kislovskiy, Y., … & Chinman, M. (2022). Getting To Implementation (GTI)-Teach: A seven-step approach for teaching the fundamentals of implementation science. *Journal of Clinical and Translational Science, 6*(1), e100. [72]	13 students enrolled. This course was offered through the University of Pittsburgh’s Institute for Clinical Research and Education (ICRE) within the Clinical and Translational Science Institute (CTSI), and was intended for students, trainees, and faculty from across the University seeking an introduction to IS	Short – 4-weeks	Online, synchronous sessions that were a mixture of didactic lectures, feedback from instructors on projects, and interactive sessions	United States	**Recruitment:** The course was advertised through the Institute of Clinical Research Education (ICRE) website and discussed during meetings of the University of Pittsburgh’s Dissemination and Implementation Science Collaborative (Pitt DISC) **Selection:** Participants registered in advance for the course, with a cap of 15 attendees
Miyamoto, K., Okamoto, R., Koide, K., & Shimodawa, M. (2024). Effect of web-based training on public health nurses’ program implementation capacity: a randomized controlled trial. *BMC Nursing*, *23*(1), 678. [76]	197 public health nurses working full time at public health offices with two to five years of experience	Short – 4 online modules that were 30 min each (2 h total)	Online, self-paced modules	Japan	**Recruitment:** To solicit participation, survey documents were sent by mail to 53 prefectural health centers, 123 health centers controlled by government-designated cities or major cities, and 138 municipal health centers in the Kansai, Chubu, and Chugoku regions of Japan in mid-October 2022. Additionally, booklets outlining this study were sent so that PHNs could apply for participation based on fully comprehending the study **Selection:** Participants were selected from PHNs working full-time at public health offices with two to five years of experience who were not on sick, maternal, or parental leave
Rakhra, A., Hooley, C., Fort, M. P., Weber, M. B., Price, L., Nguyen, H. L., … & Baumann, A. A. (2024). Training in eight low-and middle-income countries: lessons learned from a pilot study using the WHO-TDR dissemination and implementation massive open online course. *Frontiers in Health Services*, *3*, 1,217,619. [86]	247 Non-communicable diseases (NCDs) investigators in low and middle income countries (LMICs)	N/A	Online – Massive open online course	Multiple low and middle income countries (LMICs)	**Recruitment:** A recruitment email invited anyone interested in the MOOC with a brief description of the course, timeline and expectations **Selection:** There were no inclusion or exclusion criteria. Participants were invited from the Global Research on Implementation and Translation Science (GRIT) Consortium and from GRIT members networks through snowball sampling
Stevens, K. R., De La Rosa, E., Ferrer, R. L., Finley, E. P., Flores, B. E., Forgione, D. A., … & Wooten, K. C. (2021). Bootstrapping implementation research training: A successful approach for academic health centers. *Journal of Clinical and Translational Science*, 5(1), e168. [78]	63 faculty scientists and clinical partners from Texas CTSA hubs	Short –2-day workshop	Hybrid – in person 2-day training and online NIH/NCI online program as foundational knowledge for the training	United States	**Recruitment:** Faculty from all four Texas Clinical and Translational Science Awards (CTSAs) were invited **Selection:** N/A
Villemin, R., Dagenais, C., & Ridde, V. (2024). Evaluative study of a MOOC on knowledge translation in five French-speaking countries. *Plos One*, *19*(4), e0299923. [87]	In 2021, 923 people out of 2,007 indicated at the start of MOOC 1 that they intended to complete all the proposed activities and potentially also request the attestation of completion. Four months later, as of January 17, 2022, 323 people had obtained the attestation	N/A	Online – Self-paced massive open online course (MOOC) format	Several, including Canada, France, and three West African countries (Mali, Senegal, and Burkina Faso)	**Recruitment:** The MOOCs are brought to the attention of the public through their dissemination on social networks (Twitter, Facebook). The RENARD team and its partners bring together hundreds of researchers in a dozen countries around the world, all of whom have been invited to participate and relay the information **Selection:** N/A

### Location, format, & length of trainings

Table 2 describes the locations, format, and length of trainings. Of the 20 trainings, ten (50%) were conducted in the USA, four (20%) in Canada, and four (20%) in other countries (Japan, Kenya, Germany, and the UK). Two (8%) were massive open online courses available to participants in multiple countries. Five training programs (25%) were short in length, ranging from four 30-min online modules to a 4-week online program, four trainings (20%) were medium length, ranging from a summer long institute to a 9-month program. Nine were long (45%), ranging from an 18-month program to 2-year programs. There were six programs (30%) that were 2-years long, including: the Implementation Research Institute or IRI, a Master’s of Science Program in Germany, the University of Nairobi training program, the Knowledge Translation Challenge in Canada, the Mentored Training for Dissemination and Implementation Research in Cancer (MT-DIRC), and the Institute for Translational Research Education in Adolescent Drug Abuse (ITRE). Two Massive Open Online Courses did not list the length of the training. Three trainings (15%) were offered in person only, ten (50%) were offered hybrid (with both in person and virtual components), and five (25%) were offered virtually only. Two trainings (10%) did not list the format they were offered in.

### Equity focus

Only four trainings (20%) had an explicit focus on integrating equity. The detail about how different trainings conceptualized equity varied. For example, Friedman et al., 2021 noted that health equity was a focus of their CPCRN Scholars Workgroup but did not provide details. Rogal et al. [72] integrated health equity into their training by: 1) presenting a lecture by a health equity expert early in the course, 2) illustrating the Health Equity Implementation Framework as an example of an IS Framework, 3) discussing equity as a crucial aspect of proactive planning and tailoring of the evidence-based practice and implementation strategies for known disparities and barriers in priority populations and implementation contexts, and 4) emphasizing the importance of iterative and ongoing measurement and evaluation of health equity over time as an essential implementation outcome. In their 4-week online course, Rogal et al., “decided a priori to emphasize health equity and human-centered design. As such, we presented a lecture by a health equity expert early in the course and illustrated the Health Equity Implementation Framework [28] as an example of a key IS Framework. We then discussed equity as a crucial aspect of proactive planning and tailoring of the evidence-based practice and implementation strategies for known disparities and barriers (determinants) in priority populations and implementation contexts. Moreover, we emphasized the importance of iterative and ongoing measurement and evaluation of health equity over time as an essential implementation outcome that reflects the quality of sustainability capacity to adapt the “fit” of the evidence-based practice to dynamic context. In our course evaluation, we evaluated the extent to which students felt we had addressed equity.” Vroom, Albizu-Jacob, and Massey (2021) addressed equity by infusing service learning in their curriculum, which they posit is an “ideal vehicle for addressing social justice issues and health disparities because it requires extensive collaboration between academic institutions, students, and the community” [85]. Stevens et al. published monthly newsletters after the completion of the 2-day workshop that include resources on equity in IS [78].

### Competencies guiding the trainings

A total of 307 unique competencies were identified across the articles that described them. We could not identify competencies in 10 articles. For the rest of the 32 articles, we catalogued the competencies that were described by the authors. It is important to note that not all competencies were well defined. For example, some authors stated that participants were trained in “adaptation” without defining what about adaptation was being taught. That is, they did not specify whether the training aimed to identify adaptation methods or to use/apply adaptation methods. Additionally, sometimes the competencies did not distinguish between implementation and dissemination and instead were bundled as “D&I” (e.g., knowing about D&I concepts). In these instances, we double-coded the competencies for “implementation” and “dissemination” and marked them with an asterisk in the tables.

#### Different levels of competencies

Only eight articles (21%) explicitly talked about competencies at different levels of expertise with D&I. Specifically, Padek et al. [3] identified 43 D&I research competencies, which were categorized as: beginner (11 competencies), intermediate (27 competencies), and advanced (5 competencies). Participants selected the list of competencies as beginner, intermediate and advanced as ordinal numbers (i.e., 1.2.3) across four sections: (a) definition, background, and rationale, (b) theories and approaches, (c) design and analysis, and (d) practice-based considerations. To prevent unintended bias by the research team, the authors allowed participants to self-identify their skill, thus did not provide definitions for these skill levels.

Two articles used Padek’s competencies for evaluation [81], and two articles expanded and adapted Padek’s list. Rogal et al. [72] used similar competencies to develop a training program, and Heubschman et al. [7] expanded Padek et al.’s competencies to add eight competencies related to health equity and speed of translation: four competencies in emerging beginners, six intermediate competencies, and two advanced competencies. Friedman et al. [84] also developed competencies for beginner, intermediate, and advanced, and Alonge et al. [88] outlines competencies for basic awareness, beginner, intermediate, advanced, and expert. In a debate article, Mehta et al. [89] refer to the importance of Clinical and Translational Science Award programs in capacity building for early-stage faculty, mentors, consultants, and collaborators but do not specify how these competencies would differ depending on the audience. However, as with Padek et al., Tabak et al. and Mehta et al. allowed participants to self-identify their skill level, rather than providing definitions [3, 79, 89].

#### Types of competencies

Below, we describe the results from Tables 3, 4, 5, 6, 7, 8, 9, 10 and 11 with competencies grouped by the themes outlined above. Competencies were copied and pasted from the papers to allow for cataloguing (i.e., they are not our interpretation). Because papers often described more than one competency, the number of competencies does not match the sample of papers. The backslash marks a new competency. When a competency had verbs related to knowledge and to skill (see above regarding how these themes were coded), they were double coded and noted with an asterisk.

**Table 3:** General knowledge competencies

**Knowledge - General Research**
Educational knowledge*
Academic knowledge*
Program evaluation knowledge*
Management knowledge*
Research methodology knowledge*
Identification of research needs
Specific Aims/Theory
**Knowledge - Evidence based practices, policies, interventions **
Being aware of evidence resources
Knowledge of the evidence-based practice/Intervention source: know how the intervention was developed/ Knowledge and beliefs about the intervention: Have the knowledge, skills and belief required for one’s own intervention.
Determine which evidence-based interventions are worth disseminating and implementing; synthesis of all available knowledge compiled into a single harmonious statement, such as a systematic review/ Evidence strength and quality: Know the extent to which the intervention is evidence-based/ Relative advantage: Know the advantage of the intervention versus an existing project./ Cosmopolitanism: Identify interventions in other regions or by other organizations and exchange views and information thereon./ Assemble sufficient evidence of clinical intervention effectiveness and appropriate fit for a given clinical context
Describe the uses of meta-analytic methods
Identify core elements (effective ingredients) of effective interventions and recognize risks of making modifications to these.
Develop KT intervention and target them to different stakeholders
Evidence-Based Recommendations: Context and Opportunities
**Knowledge - Context **
Knowledge about the clinical practice/Make research more relevant/Setting knowledge and skills/context/consider and enhance fit/Describe factors that influence research adoption, implementation, maintenance, and reach/Assess need and context: Work with stakeholders to understand population and community needs and the extent to which potential interventions meet identified needs for particular target populations.
Knowledge about facilitating EBP in general/Be able to incorporate stakeholder input into IR practice
Knowledge of enablers and barriers in implementation/Identify barriers and facilitators
Understand multi-level context/Clarify conditions for implementation (including procedure, scope, and period).
Describe how to frame and analyze the context of D&I as a complex system with interacting parts.
Understand the role of systems in affecting healthcare and public health performance/ External policy and incentives: Identify and utilize trends in central and prefectural government policies in a timely manner.
Readiness for implementation: available resources: Identify and prepare the space and equipment for implementing the intervention./ Readiness for implementation: leadership engagement: As a leader, explain the details of the intervention to the team members and support their roles./Readiness for implementation: access to knowledge and information: Develop an environment for the intervention team members to improve their competencies (opportunities for training and provision of teaching aids, etc.).
Identify complex systems and their characteristics
**Knowledge- Health-Services research**
Knowledge of key concept
Identification of relevant themes
Knowledge of structures of healthcare system
Knowledge of outcome parameters
Knowledge of central players of healthcare system
Knowledge of in- and out- patient care structures
Knowledge of perspectives of different actors in health care
Knowledge of challenges in health care systems
Ideas for future developments in health care system

note: an asterisc (*) entails that this competency was considered as both dissemination and implementation

**Table 4:** Knowledge about D&I research

**Knowledge about D&I**
Translation into action, often referred to as evidence-based clinical practice guidelines, combining the evidential base and expertise to extend recommendations
Implementation science knowledge */ basic concepts and definitions of the science, its historic provenance/ to define the basic concepts of KT and use
Understand IS terminology/define IS terminology
Differentiate between IS research and related disciplines/Define what is versus what is not IS/Differentiate between D&I research and other related areas, such as efficacy research and effectiveness research.
Describe how IS relates to other sciences, such as improvement science and knowledge mobilisation
Identify the potential impact of IS methods
Identify the potential impact of disseminating, implementing, and sustaining effective interventions.
Identify EBPs worth implementing/the distinction between, and the evidence for, two practice-change technologies: clinical/behavior change interventions and implementation strategies
Generic presentation so applies to multiple disciplines
Implementation science: An organizational perspective
Designs and Types of Evidence for D&I Research
**Knowledge - Theories, Models, and Frameworks**
Identify appropriate conceptual models, frameworks, or program logic for D&I change/Principles and development of the theoretical approaches and models of IS
Describe a range of D&I strategies, models, and frameworks.
Map complex systems using causal flow diagrams
Determine the range of factors - behavioral, societal, ethical, institutional, political, economic, historical - that inform the research question, and design structure/contextual factors influencing implementation, such as organizational context and leadership, and the ability to understand whether a given study will observe, control, or manipulate those factors/Political & economic analysis, stakeholder assessments, cost-effectiveness
**Knowledge - Strategies**
Describe a range of IS strategies, models, and frameworks
Understanding translation and dissemination activities
Knowledge of implementation strategies
Describe a range of D&I strategies, models, and frameworks.
Knowledge about supporting change processes/understanding KT and EBP process
Develop an implementation team
**Knowledge - Methods, Designs, and Measures**
Pragmatic Trials 101
Describe participatory methods/ Articulate the strengths and weaknesses of participatory research in D&I research.
Determine when engagement in participatory research is appropriate with D&I research.
Describe study designs for IS research, including introduction to hybrid designs/ Understand the value of type 1 hybrid design in all phases of clinical research/Operationalize hybrid effectiveness-implementation designs when appropriate to accelerate the implementation of evidence-based interventions in real-world settings / Summarize study designs used in implementation research and their relative strength
Develop and assess processes and outcomes that support iterative cycles of implementation and bidirectional flow of information (e.g., learning health systems)
Describe the range of expertise needed to conduct D&I research (e.g., mixed-methods experience, economic, organizational, policy, clinical)
Identify IS measures
Knowledge of quality improvement methods and tools, communication strategies, and health policy and systems
Describe the core components of external validity and their relevance to D&I research.
Identify common D&I measures and analytic strategies relevant for your research question(s). (also in equity)
Identify and measure outcomes that matter to stakeholders, adopters, and implementers.
Describe the application and integration of mixed-method (quantitative and qualitative) approaches in D&I research.
Identify possible methods to address external validity in study design reporting and implementation.
List the potential roles of mediators and moderators in a D&I study/To identify KT mechanisms and activities
Identify and articulate the trade-offs between a variety of different study designs for D&I research.
Describe gaps in D&I measurement and critically evaluate how to fill them.
Describe how to use logic models/theories of change methodology in implementation studies
Describe how to conduct evaluation of complex health interventions
Describe how to measure successful partnerships for D&I research.
Define approaches for designing systems
Comparative effectiveness research
Compute sample size, power, and precision for comparisons of two independent samples with respect to continuous and binary outcomes.
Experimental design
Practical
**Knowledge - Adaptation and fidelity**
Describe the concept and measurement of fidelity
Describe the adaptation of an EBP/Clarify how the intervention can be modified or adjusted to meet local needs.
Understand the sources of error: fidelity/lapses in implementation as a source of reduced/heightened effect
Fit/adaptation: The capacity to control and manage organizational and community demands to ensure a balance between fit and fidelity to the critical components of the program. This focuses on the importance of recognizing the need, values, and acceptability of the EBP within the population and the capacity of the agency against the critical requirements and components of the program in question. This may include the capacity to make meaningful adaptations when necessary to increase the fit and acceptability for the organization and/or population of interest./Consider and enhance fit
Characterize process models that support iterative cycles of implementation and adaptation based on learning
Describe how adaptations will be documented throughout the D&I research project
Balancing Fidelity and Adaptation: If We Want More Evidence-Based Practice, We Need More Practice-Based Evidence
**Knowledge - Dissemination**
Describe a process for designing for dissemination (planning for adoption, implementation, and sustainability during the intervention development stage)/Knowledge, dissemination, translation and diffusion research
Describe whether a study is a dissemination study
Dissemination science
**Knowledge - De-implementation**
Identify potential impact of de-implementation
Identify the potential impact of scaling down (aka de-implementing) an ineffective but often used intervention.
**Knowledge - Ethics**
Knowledge of ethical and legal guidelines
Responsible conduct of research and implementation
Summarize the importance of ethically and culturally competent clinical and community-based research in D&I science
Identify potential ethical issues in IR, such as safety of participants, power relationships, literacy, disruption of services

note: an asterisc (*) entails that this competency was considered as both dissemination and implementation

**Table 5:** Skills competencies about research in general

**Skills - Research in general**
Communication skills*/sharing knowledge/communicating research findings/success in research dissemination to appropriate audiences
Educational skills*
Academic skills *
Program evaluation skills*
Management skills*/research conduct and/or management strategies
Research methodology skills*
Data management, analysis, and visualization; SPSS
Knowledge synthesis
Use of research fundings (or research use)
Develop research methods and measures
Experience in handling different data source and routine data
Experience in planning a research study/end-of-grant KT
Ability to develop an evaluation plan
Knowledge of criteria for scientific integrity and fidelity
Project organization
Oral presentations for an academic audience
Literature search/Identify relevant theory, evidence, methods, and perspectives outside the clinical domain of the research program/Summary/review of research literature; analysis and synthesis of results
Ability to write an academic report
Ability to write grants and publications among translational researchers, ability to write manuscripts in general
New, marketable discoveries
Ability to write IRB protocols
**Skills - Intervention design**
Integrate diverse disciplinary, stakeholder and community perspectives into a cogent intervention design and/or implementation and dissemination strategy
Utilize a comprehensive implementation framework to guide the integration of theory with specific intervention, evaluation, and dissemination activities
Developing an intervention
Self-efficacy: Have belief in one’s own capabilities/a sense of self-sufficiency in implementing the intervention.
Individual stage of change: Be prepared to implement eachphase of the intervention on one’s own knowledge/persuasion/decision/ execution/confirmation)
Develop a logic model for designing evidence-based, theory-driven program

note: an asterisc (*) entails that this competency was considered as both dissemination and implementation

**Table 6:** Skills competencies related to D&I research

**Skills - D&I science **
Realist Synthesis: Building the Evidence Base for D&I Research
Identify existing gaps in D&I research/formulate methods to address barriers to address D&I research; capacity to conduct synthesis to address KT questions
Explain the evolution, current state, and future agenda of implementation science and its value to population health
Implementation science skills*/opportunity for participants to apply basisc of KT in their own settings/State a research question addressing a gap in the provision of an evidence-based intervention, practice, or policy
Identify and recruit sites for IS research/ Identify sites to participate in D&I studies, and negotiate or provide incentives to secure their involvement./ identify the practice change they want to implement, document the need for this change in their practice context, and summarize evidence of its effectiveness
Employ evidence-based practice/Implementation/ Practical application of IS in implementation research
Ability to articulate implementation science as an innovative approach to clinical and community-based research
Link barriers and facilitators to behavior change theory
Application of IS in specific contexts (e.g., LMICs)
Determine which evidence-based interventions are worth disseminating and implementing
**Skills - Context**
Assess, describe, and quantify (where possible) the context for effective D&I (setting characteristics, culture, capacity, and readiness)/must be able to determine which issues are most central to consider for their agency
Develop context-specific implementation strategies for scaling up best-practice intervention
**Skills – Theories, Methods and Frameworks**
Ability to select, critique, and use an established conceptual model or framework to guide a research study/TMFs knowledge, skills, and self-efficacy in KT intervention development and implementation/Use an evaluation framework to guide evaluation/Select conceptual models and theoretical justification to support the choice of implementation strategy and inform the design, variables to be measured, analytic plan, and sustainment
Determine which approach(es) have the highest potential to produce successful implementation in their unique service setting
**Focus on impactful outcomes: translational and societal outcomes**
Provide clinical and translational science instruction to beginning scientists
Integration into practice is evidence-in-action, in which practice is aligned to reflect best evidence
Incorporation of discoveries from bench science into human or animal studies/Translating research to practice
Incorporation of clinical trial results into clinical or practical guidelines/Assess data sources and data quality to answer specific clinical or translational research questions
Improvement in evidence-based health care service and delivery, patient outcomes and positive health behavior/ Clarify the need to implement a new intervention in response to the trend of health issues
Derive translational questions from clinical research data/ Interpret published literature in a causal framework./Horizontal development of Pioneering and superior practice: Identify advanced good practices and their implementation in other regions or by other organizations
Formulate well-defined clinical or translational research question
Identify basic and preclinical studies that are potential testable clinical research hypotheses
Integrate elements of translational research into given study designs that could provide the bases for future research, such as the collection of biological specimens nested studies and the development of community-based interventions
Propose study designs for addressing a clinical or translational research question
Implementation outcomes, inputs, strategies/ Define outcome measures for both implementation strategy (system outcomes) and clinical intervention (patient/population outcome)
Confirmation of higher goals/incentives: Confirm consistency with higher goals (such as comprehensive plan or basic guidelines)
Inclusive view of the impact that the evidence-based practice has on patient health outcomes; satisfaction; efficacy and efficiency of care; and health policy
Strengthening and refining health related policies and procedures
Improvement in community health, empowerment, and economic conditions to reduce health disparities
Development of new community-based participatory programs and partnerships geared toward effective and meaningful implementation
A Rapid-Learning Healthcare System: Using Research, Adopting Best Practices/Examine the importance of rapid research to advance D&I science concepts and directions
Work with partners to select IS outcomes measures
Global Applications of Implementation Research
**Skills - Methods and Designs**
Comparative Effectiveness Research: Moving the Field Ahead and Disseminating Results
Describe how to apply participatory methods in IS/ Be familiar with user-centered design; making interventions useful, usable, and desirable (design for dissemination)
Apply common D&I measures and analytic strategies relevant for your research question(s) within your model/framework/the multi-level nature of practice or service system change, and ability to select appropriate research methodologies, including randomized control designs and alternatives to group randomization/Selection of appropriate methods designs/Formulate methods to address barriers of D&I research./Describe implementation strategies for moving evidence into practice including existing taxonomies/classification schema
Practice-Based Research Networks: Participatory Laboratories for Discovery, D&I
Measure partnerships for IS research
Assess readiness for change in implementation settings
Evaluate and refine implementation strategies/measurement of implementation strategies and outcomes
Assess threats to internal validity in any planned or completed clinical or translational study, including selection bias, misclassification, and confounding
Assess threats to study validity(bias) including problems with sampling, recruitment, randomization, and comparability of study groups
Understand the aims of process, outcomes and impact evaluations and the application of these evaluation types in implementation
Select appropriate evaluation indicators/Design a research data analysis plan
Expertise in evaluation of implementation plans/Assess and mitigate risks in design/capacity to design and evaluate the impact, effectiveness, and sustainability of KT strategies in different settings/develop an implementation plan/Assess the implementation context
Integrate mixed methods in IS research/Gain facility with qualitative and quantitative experimental designs to plan, implement, and evaluate interventions and policy impact/Qualitative and quantitative research methods: paradigms, design, implementation, data analysis, writing/capacity in multiple research methods including qualitative methods to examine the determinants of knowledge use across different settings and stakeholder groups/Practical experience in quantitative surveys and descriptives analysis/Experience in conducting qualitative interviews
Evaluation of applied research methods/multilevel modeling
Design and Evaluation of study protocols
Evaluate implementation quality
Design and prototype service delivery processes and systems
Determine and measure processes and outcomes that support iterative cycles of implementation and bidirectional flow of information/Develop and assess processes and outcomes that support iterative cycles of implementation and bidirectional flow of information (e.g., learning health systems)
Alternative sources of data for implementation research
System Dynamics Tools for D&I/ Apply systems science and systems modeling approaches in D&I research
Examine the importance of rapid research to advance D&I science concepts and directions
In Search of Synergy: Strategies for Combining
Interventions at multiple levels
Designing, Measurement and Evaluation
Understand key constructs of participatory research, that is, collaborative, equitable, community-based, co-learning, capacity building and so on/Summarize(analysis level) the principles and practices of the spectrum of community-engaged research
**Skills - Adaptation and fidelity**
Identify a process for adapting an intervention in IS research/Identify and explore adaptive challenges to implementation
Explain fidelity and adaptation when developing interventions/strategies
Explain how to maintain fidelity of original interventions during the adaption process
Tailor strategies/Tailor implementation strategies to the implementation context
Use evidence to evaluate and adapt D&I strategies for specific populations, settings, contexts, resources, and/or capacities
Appropriate adaptation of context relevant interventions
Evaluate adaptations and their potential impact on outcomes
Understand the values, loyalties, losses, and benefits of different approaches to a variety of adaptive challenges
Characterize process models that support iterative cycles of implementation and adaptation based on learning
**Skills - Dissemination**
Dissemination of research findings/Communicate complex information to various end-user groups in appropriate formats, considering suitability and readability of content
Sharing knowledge
Fostering innovation/making research more relevant
Knowledge brokering
Strengthen communication skills
Disseminate research/program results to relevant stakeholders and communities in a manner that maximizes their influence and sustainability outside of the research paradigm
Plan for and carry out dissemination initiatives by selecting key messages and appropriate dissemination strategies
Develop knowledge tools for various end-user groups/Disseminate to people on the ground
Designing for Dissemination
**Skills - De-implementation**
Explain de-implementation in IS/Effectively explain and incorporate concepts of de-adoption and de-implementation into D&I study design
**Skills - Sustainability and scale up**
Integrate sustainability plans and concepts into work/Effectively integrate the concepts of sustainability/sustainment and the rationale behind them in D&I study design
Scale-up and spread/Develop sustainability IS partnerships/Identify and develop sustainable partnerships for D&I research./understand factors related to sustainability, spread and scale-up of evidence based practices outlined in various theories and frameworks
Understand methods of scaling up and what is required for each/Evaluate and refine innovative scale-up and spread methods (e.g., technical assistance, interactive systems, novel incentives, and “pull” strategies)
Develop a sustainability plan/Ensuring continuity between funding, partner cycles/ Describe key elements in forming a business plan for sustainment, identifying implementation costs and quantifying benefits
Sustainability
Create implementation support for spread and scale up
Marshal resources to ensure continuation and continuity of the supports necessary for sustainability/The capacity to sustain the supports necessary to ensure the ongoing success of the program. This will likely include maintaining the resources (e.g., monetary and/or personnel), required for the program, ancillary support services, and integrating the program into regular business practices
**Skills - Ethics**
Understanding of and ability to address the specific ethical issues in IR and capacity for responsible conduct of research
Discuss the cultural and social variation in standards of research integrity
Apply the main rules, guidelines, codes, and professional standards for the conduct of clinical and translational research

note: an asterisc (*) entails that this competency was considered as both dissemination and implementation

**Table 7:** Competencies related to engaging with other disciplines

Employ epidemiological methods in study designs, program evaluations and causal inference
Apply and integrate implementation science approaches: Apply and integrate appropriate implementation frameworks, models and strategies by using systems thinking, participatory methods, and knowledge management and exchange
Understand the importance of value proposition, designing for dissemination, cost effectiveness, and policy implications
Policy Dissemination Research/Practical application of IS in policymaking
External policy: The capacity to remain informed and act on external policy, mandates, and recommendations and guidelines on the local, state, and federal levels that have the potential to facilitate and/or hinder the implementation and maintenance of a new intervention
Explain how knowledge from disciplines outside of health (e.g., business, marketing, and engineering) can help inform further transdisciplinary efforts in D&I research
Identify and articulate the interplay between policy and organizational processes in D&I/Policy-to-programming, development of innovative approaches to improve healthcare delivery
Describe the range of expertise needed to conduct D&I research (e.g., mixed method experience, economic, organizational, policy, clinical)
Describe the relationships between various organizational dimensions (e.g., climate, culture) and D&I research
Incorporate economic evaluation into IS work/Incorporate methods of economic evaluation (e.g., implementation costs, cost-effectiveness) in D&I study design/ Qualify the costs associated with implementing the intervention by expense item
Employ weighted evidence, cost-effectiveness, and translation into policy
Work together with scholars from different disciplines
Collaborate with bioinformatics specialists in the design, development, and implementation of research projects
Utilize informatics/ Communicate, manage knowledge, mitigate error, and support decision making using information technology
Develop protocols utilizing management of information using computer technology
Describe the effects of technology on medical research, education, and patient care
Apply theory and strategies from team science to promote team effectiveness in D&I research/Apply principles of the “science of team science” to enhance productivity of multidisciplinary study teams and achieve adaptive implementation andsustainable change
how implementation science could be applied to multiple health and social service disciplines including occupational therapy, nursing, public health, social work, rehabilitation and mental health counseling, anthropology, and education
Basic knowledge in epidemiology
Knowledge in health economics
Knowledge in organizational development
Knowledge in business administration
Knowledge of data sources in health reporting
Knowledge of quality management in healthcare
Knowledge of data sources for quality assessments
Apply quality improvement

**Table 8:** Competencies related to equity

**Engaging the community**
Understand the stakeholders that should be engaged
Understand the value of early engagement of stakeholders/End-user knowledge and experience being valued equally with that of professionals/ Outline an engagement process that will gain support from relevant stakeholders to ensure feasibility of the study plan
Ensure research is meaningful/Equity in relationship building: Prioritize questions with high relevance to stakeholders/assess fit for their population
Participatory Approaches: How Can CBPR GuideTranslation and Dissemination?
Be able to engage stakeholder groups appropriately to gather perspectives and opinions/improve practice partnerships/Describe the importance of incorporating partners /Describe the importance of incorporating the perspectives of different stakeholder groups (e.g., patient/family, employers, payers, healthcare settings, public organizations, community, and policy makers)./Build relationships with community members and community-based organizations in order to engage multiple perspectives on the problem/skills in engaging relevant stakeholders (including the public, healthcare providers, managers, and policy makers) to facilitate an integrated KT approach/stakeholder engagement/Engage (or encourage others to engage) in action planning to resolve anticipated and unanticipated implementation issues/ Understand the benefit of and how to communicate with relevant stakeholders/Brokering: Enable knowledge exchange and sharing among stakeholders to increase understanding of diverse perspectives and increase the application of implementation science to improve outcomes/integrate strategies within D&I research to facilitate meaningful stakeholder engagement (e.g., shared power, shared decision-making, co-learning)/ Engaging: intervention participants: Recruit intervention participants via multiple publication media/channels
Describe the appropriate process for eliciting input from community-based practitioners for adapting an intervention./Know methods of engaging and involving stakeholders at all key points of ETP implementation (planning, implementation, evaluation, sustainability), to incorporate stakeholder interests in the process
Identify all key stakeholders, define the nature of their stake in the change and determine their level of buy-in for the change
Integrate strategies within D&I research to facilitate meaningful stakeholder engagement (e.g., shared power, shared decision-making, co-learning)
**Methods and Approaches**
External Validity: Why it Matters/ Understand the relevance of study design and choice of target group to external validity and ultimate translatability.
Be aware of models and methods for facilitating stakeholders' engagement and participation
Identify and apply techniques for stakeholder analysis and engagement when implementing evidence-based practices/ Understand how to identify relevant nonacademic stakeholders in research and how and when to engage with them to aid in movement across research stages and translation into practice/Apprise (analysis & evaluation levels) the role of community engagement as a strategy for identifying community health issues, translating health research to communities and reducing health disparities/Engage stakeholders
Apply methods to find patient and community needs
Develop design requirements from needs
Co-design: Co-design tools, resources, and models through participatory, iterative processes and consensus building
Explain the special issues that arise in research with vulnerable participants and the need for additional safeguards
Scaling community models
Design strategies to address the multi-level influences of health inequities as it relates to the implementation of an evidence-based intervention
Be able to strategise to address inequities in implementation/Describe studies that recognize the determinants of health disparities
Identify the potential impact of disseminating, implementing, and sustaining effective interventions, including assessments of equity and representativeness
Develop strategies to promote equity in resource distribution across all external research partners, including community partners or other external organizations and the researcher's institution
**Health Literacy and Cultural Competency**
Discuss the role of health literacy principles of human subjects in their ability to learn, retrain, and practice health information
Specify (synthesis level) how cultural and linguistic competence and health literacy have an impact on the conduct of community engaged research.
Differentiate between cultural and population diversity principles.
Summarize the importance of ethically and culturally competent clinical and community-based research in D&I science

**Table 9:** Competencies related to attitudes and relational aspects

Collaboration and teamwork/work in interdisciplinary teams/develop a collaborative, multidisciplinary team that shares a common language, and promotes a transdisciplinary blending of disciplines/Communication, teamwork, collaboration/ Networks and communications: Hold meetings to consult on implementation and secure communication tools such as e-mail and telephone
Collaboration/communication (both internal and external): The capacity to build and maintain collaborations and communication channels among required partners. Internally, this may include leadership debriefing with staff and providing ample opportunity and support for inter-organization collaboration as well as organizations communicating goals and visions to its staff and/or instituting formal internal policies to ensure support of the organization’s mission can be fulfilled. Externally, multiple service organizations may be in communication with one another with the intention to share insight on the implementation process./ Culture: Take into account the impact of organizational culture (including norms, values, and characteristics)
Engage in collaborative writing, including the production of grants and manuscripts that meet the unique needs of sponsors of implementation and dissemination science
Leadership/The capacity to provide dedicated leadership to the implementation, integration, and support of the new program. This may entail new leadership structures, reassignment of positions or lines of authority, and empowering decision-making and supervisory responsibilities./Work as a leader of a multidisciplinary research team./Leadership, behavior change, organizational culture/ Fostering change acceptance climate: Ensure that the organization recognizes and accepts the priority and importance of the new intervention
Implementation climate: learning: Ensure that the organization develops a culture and system to gain knowledge and skills required for the intervention
Collaboration knowledge and skills
Challenges of interprofessional collaboration
Oral presentation skills for a public audience
Build an interdisciplinary/ intradisciplinary/multidisciplinary team that matches the objectives of the research problem
Improve practice partnerships
Describe the importance of incorporating organizational partner perspective
Ability to identify conflicts of interest
Provide patient-centered care
Frank/direct/honest
Professional
Creative/flexible/innovative/adaptive
Motivated/motivating/encouraging/empowering
Authentic
Empathetic/respectful/sensitive
Collaborative/inclusive; communication/collaboration
Confidence
Grow and sustain relationships: Grow and sustain diverse, authentic, respectful and trust ing relationships with stakeholders to guide and support implementation and systems change efforts
Having trust; Intrapersonal trust (the belief that the implementation support practitioner is reliable, competent, and committed to the change effort on behalf of the organization they are supporting); Interpersonal trust (the perception of both implementation support practitioners and their stakeholders that they are in a collaborative and reciprocal relationship focused on achieving identical aims)
Valuing research
Self-directed lifelong commitment to learning
Skills related to KT planning, project management, information technology use, sound judgment, discretion tact, diplomacy and resourcefulness/Manage a clinical and/or translational research study/learn to collaborate with team
Integrity, commitment to professional work ethic and behavior in interaction with contracts, commitment to high standards of professionalism, and interest in the latest developments in communications
self-awareness
self-management
social awareness
Reflexivity: Researchers (and other partners) strive to be aware of and analyze how their positions may influence the collaborative’s dynamics
Reciprocity & mutuality: Partners are interested in learning from each other. Relationships are perceived and experienced as mutually beneficial through the combined knowledge and the deepened networks developed/Co-learning: Work collaboratively with stakeholders to learn how applied knowledge on implementation science can be effectively used in local contexts
Transformative & personalized: The collaborative process benefits the study while also offering an enriching individual experience through use-value and empathy
Relationships facilitated: Relationship structures and procedures are developed collectively to support the implementation collaborative
Address power differentials: Address power imbalances between stakeholders by building trust, supporting two-way communication, and cultivating opportunities for mutual consultation
Facilitation: Enable participatory problem solving and support in a context of a recognized need for improvement and supportive interpersonal relationships
Use the soft system approach to address messy problems
Capability – the psychological and physical capacity to initiate behavior change [note: considered as an outcome by Albers et al]
Motivation in improved attitudes towards using evidenced implementation concepts [note: considered as an outcome by Albers et al]
Opportunity – factors enabling or prompting an intended implementation behavior – in changes to the organizational climate surrounding an implementation [note: considered as an outcome by Albers et al] effort
Mediate between different interests of stakeholders using skills such as team building, negotiation, conflict management and group facilitation to build partnerships in pursuit of a common goal
Tailored support: Determine frequency, duration and intensity of implementation supports based on the needs, goals and context of the implementation team and systems stakeholders
Develop strategies for overcoming stakeholder ambivalence or resistance to change
Exhibit interpersonal communication skills that demonstrate respect for other perspectives and cultures

**Table 10:** Competencies related to capacity building

Build capacity for research
Integrating IS into programs/ research (rather than add-on)
Build capacity: Build the knowledge, skills, and motivation of stakeholders to achieve their goals. Pay attention to different capacities (psychological, behavioral, structural, innovation-specific, general, analytic, adaptative) at all levels of the system (individual, organization, network, and system).
Being able to do the actual work of implementing the EBP through staff training, instituting new policies, and negotiating new contracts
Education/training/coaching: The capacity to provide ongoing training and education both during implementation and later for sustaining the new program. This may include periodic retraining, as well as onboarding new staff and acquiring train the trainer opportunities.
Acquire and/or allocated resources: The capacity to acquire the funding and resources necessary to adopt, implement, and sustain new programming. This may involve funding from the local, state, and/or Federal levels and resources such as physical space, technology, education, and time./Develop strategies to promote equity in resource distribution across all external research partners, including community partners or other external organizations and the researcher’s institution/Draft a prospectus targeted at a D&I funding opportunity from a variety of agencies
D&I Funding Opportunities
Implementation/organizational readiness: The capacity to develop or build organizational readiness for the new program. This may involve reviewing, documenting, and modifying policies and procedures, increasing program awareness, and can include identifying and addressing indicators of organizational commitment to implement the new intervention
Organizational culture and climate: The capacity to identify and change organizational culture (underlying beliefs, assumptions, and missions/values that contribute to the environment of an organization) and organizational climate (shared perceptions of the psychological impact of the work environment on the employee)./Goals setting and accountability: Ensure that the organization sets and publishes the goals to be achieved by the intervention.
Engaging: internal implementation key persons: Place key persons in supervising/ directing positions and the execution team.
Engaging: external change agents/ key stakeholders: Partner/collaborate as necessary with relevant external parties and organizations.
How to introduce IS to junior scientists
How to be involved in the world of IS
How to access IS information
Develop strategies that strengthen community capabilities for overcoming barriers to health and well-being
Assist host entity in assessing existing capacity

**Table 11:** Competencies related to quality improvement

Identify errors and hazards in care, understand and implement basic safety design principles; continually understand and measure quality of care
Conduct a situation analysis across a range of cultural, economic, and health contexts
Develop monitoring and evaluation frameworks to assess programs/Using data for implementation
Data-based decision-making and evaluation: The capacity to collect and utilize data coming from monitoring and evaluation activities to make decisions regarding service implementation. This may include monitoring fidelity and acquiring feedback from implementers about the progress of EBP implementation/How to measure if implementing optimally; Making data-informed decisions
Design context-specific health interventions based upon situation analysis
Design program work plans based on logic models/ Planning: Ensure that the organization rigorously develops a feasible execution plan/Plan an evaluation of KT impact.
Apply scientific evidence throughout program planning, implementation, and evaluation/Plan a KT process
Conduct improvement cycles: Continuously use data to purposefully re-examine implementation processes and improve practice, organization and system changes/ Reflecting and evaluating: Regularly review the progress of execution for evaluation and improvement.

We separated knowledge into two main sub-themes: general knowledge (i.e., not specific to D&), and D&I knowledge. Table 3 shows the 28 competencies of general knowledge, divided into: general research (6 competencies), evidence-based practices, policies, guidelines or interventions (7 competencies), context (6 competencies), and health services research (9 competencies). Table 4 shows the 59 competencies related to: knowledge about D&I (11 competencies), theories, models and frameworks (4 competencies), strategies (6 competencies), methods and designs (22 competencies), adaptation and fidelity (7 competencies), dissemination (3 competencies), de-implementation (2 competencies), and ethics (4 competencies).

Table 5 shows the 24 skills competencies not specifically related to D&I, divided into skills about research (20 competencies) and skills about intervention design (4 competencies). Table 6 shows the 79 skills related to D&I: general D&I skills (9 competencies), skills about assessing context (2 competencies), skills related to applying theories, methods and frameworks (2 competencies), skills related to identifying and targeting impactful translational and societal outcomes (17 competencies), skills related to methods and designs (22 competencies), skills related to adaptation science (8 competencies), dissemination (9 competencies), de-implementation (1 competency), sustainability and scale up (6 competencies), and ethics (3 skills). Table 7 outlines the 25 competencies related to “Engagement with other disciplines”, involving actions such as incorporating other fields (e.g., economic evaluation or organizational theories) in D&I research. Table 8 outlines the 24 competencies catalogued in the “Equity” related to engaging the community (9 competencies), methods and approaches (11 competencies), and health literacy and cultural competency (4 competencies). Table 9 outlines the 41 competencies related to “Attitude and Relational Aspects”. Table 10 shows the 13 competencies related to Capacity Building; Table 11 shows the 8 competencies related to “Quality Improvement”; and Table 12 shows the 6 competencies related to Mentoring.

**Table 12:** Competencies related to mentoring

Individual developmental plans, interactive case discussions/guidance on how to formulate research questions and implement or conduct studies
Strategies and tactics for effective mentorship
Maintain skills as mentor and mentee
Mentorship on how to navigate relationship with community
Mentorship on soft skills
Incorporate adult learning principles and mentoring strategies into interactions with beginning scientists and scholars in order to engage them in clinical and translational research.

Table 13 shows that the frequency of the competencies across themes varied: attitudes and relational aspects were the competencies most cited across the papers, with skills about how to apply D&I methods being the second, and knowledge about D&I methods being the third. The least cited competences in the papers reviewed included D&I ethics, knowledge about de-implementation, and intervention design skills. We did not see any trends of competencies across the years.

**Table 13:** Frequency of competencies

Competencies Themes	Frequency
**Knowledge about research in general**	
General Research	7
Evidence based practices, policies, interventions	21
Context	17
Health-Services research	10
**Knowledge about D&I research**	
Introduction to D&I	26
Theories, Models and Frameworks	24
Strategies	9
Methods, Designs, and Measures	51
Adaptation and Fidelity	12
Dissemination	6
De-Implementation	4
Ethics	5
**Skills about research in general**	
Research in general	36
Intervention design	4
**Skills about D&I Skills**	
Skills about D&I science	23
Context	5
Theories, Models and Frameworks	3
Focus on impactful outcomes: societal impacts	26
Methods and Designs	49
Adaptation and Fidelity	17
Dissemination	15
De-implementation	4
Sustainability	21
Ethics	3
**Engage with other disciplines**	41
**Equity**	
Engaging Community	28
Design and Methods	20
Health Literacy and Cultural Competency	4
**Attitudes; relational aspects**	68
**Capacity building**	17
**Quality Improvement**	12
**Mentorship**	10

## Discussion

This mapping review sought to describe the state of D&I capacity building initiatives. Our data show that, while several initiatives have been published, the literature shows a lack of consistency in the definition of competencies, in the types of competencies used to guide the capacity building initiatives, and in the evaluation of the trainings.

The data in this review show that less than half of the trainings in this study reported using a framework to design their training. The goal of this paper is not to recommend a framework to guide the development and evaluation of capacity building initiatives. We can hypothesize that the absence of a larger framework to guide the different capacity building initiatives reflects how relatively young the field is, and how it is still evolving. However, similar to the argument posed by several scholars about the appropriate selection, adaptation, use and testing of frameworks to guide research in the field of D&I [90], we can argue that frameworks can guide the development and evaluation of capacity building initiatives and provide some alignment in terms of skills and knowledge about the science in the field. Different frameworks can be used to develop a capacity building initiative versus to evaluate it. For example, the Implementation Research Institute used a combination of theoretical perspectives to guide its development, and more recently, the institute has used the Translational Science Benefit Model to evaluate the impact of the work from its alumni [54, 91, 92]. Alternatively, Miyamoto et al. [76] used the Consolidated Framework for Implementation Research to guide content development for their Capacity Development Training Course for Evidence-based Program Implementation (“EPI-TRE”) training [93]. CFIR guided the Implementation Degree Assessment Sheet (IDAS), a teaching aid that then was used as a measure for evaluating their training [94].

The granularity of the competencies used to guide the development and evaluation of the capacity building initiatives may depend on the framework, the audience, the length of training, and the goal of the training. For example, Miyamoto et al. [76] aimed to train their participants in setting up different aspects of readiness to implement the intervention (e.g., availability of resources, leadership engagement, access to knowledge and information), but other trainings simply would refer to this competency as examining contextual factors. While a common framework to guide the development and evaluation of capacity building initiatives is not necessarily an appropriate recommendation (as different initiatives can have different goals depending on their audience and funding), we propose that explicitly outlining which theoretical underpinnings inform the development and evaluation of the different capacity building initiatives can help in the identification of core competencies across initiatives and identify what works for whom as we grow the workforce in the fields of D&I.

Most of the capacity-building initiatives identified in our sample were offered in the United States, and in academic settings. Furthermore, 38% of the trainings were only offered to researchers, and no trainings were offered only to practitioners or lay persons. None of the capacity-building initiatives were in community settings, reducing the accessibility of D&I training for this audience. A discussion about the benefits and challenges of including community members as either community researchers (laypersons employed to conduct research activities in their own communities) [95], as implementers or members of research initiatives [96, 97] is beyond the scope of this study, but one could argue for the importance of engaging community members to inform D&I capacity building to help inform how to connect community with research enterprise and cross-learning. The process by which this type of capacity building would be done, however, is yet to be developed as we did not see any discussion of engaging community members as community advisory boards or as part of the capacity building initiatives in our sample of papers. If we propose that the fields of D&I decrease the quality gap and the science and research gap before they widen [98], we may need to increase the communication and shared skills between researchers, practitioners, intermediaries (i.e., people who translate findings from D&I field to support in the implementation of evidence-based practices), policy makers, educators, and leaders [38, 99]. However, the capacity building initiatives for each of these audiences may have different goals and formats, and much needs to be explored in this space.

Only about 20% (*n* = 8) of the identified articles referred to different levels of competencies, such as beginning, intermediate, advanced, and expert competencies. While we agree with Padek et al. [3] that pre-defining these learning categories may yield bias from the research team, or those developing capacity-building courses, we feel that there is merit to Alonge et al.’s [88] comment that not defining different competency levels may yield confusion in the fields. It is important to highlight that only five competencies were identified as at the ‘advanced’ level by Padek et al. [3]: describe gaps in D&I measurement and critically evaluate how to fill them; effectively explain and incorporate concepts of de-adoption and de-implementation into D&I study design; incorporate methods of economic evaluation (e.g., implementation costs, cost-effectiveness) in D&I study design; evaluate and refine innovative scale-up and spread methods (e.g., technical assistance, interactive systems, novel incentives, and “pull” strategies); and use evidence to evaluate and adapt D&I strategies for specific populations, settings, contexts, resources, and/or capacities. Recently, seven competencies were added by Heubschman et al. [7], with only two being advanced competencies.

The challenge of doing an in-depth analysis on how these advanced competencies can be addressed in the capacity building initiatives, however, is that these are self-reported classifications and evaluated in initiatives aimed for researchers. One could hypothesize that advanced competencies in D&I are related to the skills of applying the knowledge in either developing a research protocol and/or applying the knowledge in the field implementing an intervention, program, policy or guideline. To foster advanced competencies, variables such as: participants’ background knowledge of D&I and of research, the goal of the training, and the length of the capacity building initiative (e.g., one day workshop, two years) may be variables that developers of capacity building programs may need to consider. Additionally, as the fields mature, we will need to examine whether capacity-building programs identify more advanced competencies. Different audiences (e.g., researcher, practitioners, public health, healthcare leaders) could then position themselves in this continuum of competencies, depending on their goal and level of engagement with the D&I fields.

One potential proposal for the D&I fields is to conceptualize the types of competencies for the different audiences based on knowledge, skills, and other activities that their roles will require. For example, recently the medical field has advocated for developing a curriculum based not only on competencies but also on entrustable professional activities (EPAs), which are “units of professional practice (tasks or bundles of tasks) that can be fully entrusted to an individual, once they have demonstrated the necessary competence to execute them unsupervised” [100, 101] We could speculate that, if we return to the potential different levels of engagement with the D&I fields articulated in the introduction, the scientific collaborator interested in a basic level of knowledge may only need to foster knowledge competencies, the scientist who uses D&I in his/her/their research will need knowledge and skills competencies, and the expert methodologist who seeks to advance the D&I fields would strive for entrustable professional skills. That is, the scientist using D&I would be focused more on the translational aspect of D&I (translate findings from their own research into clinical or community settings), and the expert methodologist would focus on advancing the methodology. We did not find such discussion in the papers from our sample.

Our review demonstrated a lack of clarity in the definition of competencies, with three main issues. First, usually the competencies referred to implementing (or de-implementing, or developing) an evidence-based intervention within healthcare settings. Because the fields of D&I are broader than implementing only interventions in healthcare, we advocate that competencies should include attention to implementing evidence-based practices, policies, guidelines and practices in settings outside health care. Our review was unable to determine whether capacity-building programs are extending these competencies to innovations beyond the healthcare system. Second, there was inconsistent use of verbs (i.e., actions required to demonstrate the competency) in the description of the competencies. To move the fields forward, we propose that capacity building leaders be clear about the intention of the competencies. For example, if competencies are related to building knowledge, verbs such as “characterizing” or “identifying” should be used. If, however, the training aims to foster skill competencies, we suggest using the verbs “employ” or “apply”. Third, the competencies were often bundled (e.g., “learn D&I principles”). As the fields of translational, dissemination, and implementation research continue to grow, it may be important to disentangle these competencies to clarify the training goals more precisely. As we advance the science of these themes (e.g., identify mechanisms of implementation strategies or advance the science of adaptation), we could hypothesize that competencies may need to be further described as scholars become methodological experts in these areas.

Interestingly, some capacity-building programs fostered competencies related to research in general. We decided to report the competencies around general research knowledge and skills here because, depending on the goal of the capacity-building program, training in these foundational research skills may be important. For example, trainings such as IRI, MT-DIRC, and TIDHR select participants who have pre-requisite experience and funding in research [49, 54, 81]. Other capacity-building initiatives, however, especially those in graduate programs may not have an audience with expertise in general research (e.g., grant writing). Here, there would be (at least) three paths. One is to train the audience in general research skills in other courses so that – if the goal is to apply D&I science in research – they are well-equipped to do so. Second, if the training is aimed towards practitioners, for example, they may not necessarily need to be well-versed in general research skills. A third path is what may be happening in contexts where consultants sometimes provide mentoring/consultation in both research skills and D&I projects [8, 102]. As the fields of D&I grows and we develop different capacity-building opportunities, it will be important to think critically about which competencies are needed for diverse audiences (e.g., researcher, practitioners, graduate students, policy makers, public health practitioners) and which path is relevant for the type of engagement with the fields.

Several competencies were coded “Attitudes and Relational Aspects.” The literature on D&I practitioners has recognized the importance of attitudes and relational aspects [103, 104] for these professionals’ applied work. While recognizing attitudes and relationships has been more prominent in the practitioner literature, scholars have also identified the importance of relationships, self-reflection, and humility in the research space [105]. Another theme that emerged was the importance of “Engaging with Other Disciplines”. Here, there seems to be at least a three-way approach: for D&I scholars to learn from other fields (e.g., learn how to do cost analysis from economists), to apply D&I in other fields (e.g., how to use D&I methods to increase uptake of cancer guidelines), and/or to create bi-directional learnings between fields (e.g., how the cultural adaptation field can learn from implementation science and vice versa).

While the D&I fields have recently given more attention to health equity [12, 15, 106, 107], only four trainings had an explicit focus on integrating equity. As capacity-building initiatives start to incorporate more equity considerations in their trainings, it would be important to learn from and incorporate the expertise that race scholars, healthcare and social justice scholars, and psychologists who have been developing cultural competency and other trainings to incorporate attention to social justice and equity [108–110]. There were few competencies identified in “Capacity Building” and in “Mentoring.” Knowing how to gather funding to support the sustainment of initiatives and how to foster capacity building in community settings are skills that could perhaps be more explicit in capacity training initiatives. Similarly, mentoring is a key aspect of trainees’ success and should not be undervalued, as is shown in the evaluation of capacity building initiatives [54, 81].

### Challenges related to competency-based initiatives

This paper illuminates the challenges in basing D&I capacity building on competencies without a well-defined method and theory to inform capacity building efforts. We are at a pivotal moment as the fields continues to build capacity building initiatives for different audiences and for different levels of engagement with the sciences.

This study was developed with the assumption that competencies can provide a map to inform initiatives for capacity-building in D&I, as outlining competencies can inform the goals, process and evaluation of capacity building initiatives. However, it is important to note that scholars from other disciplines (e.g., psychology, education, organization) have offered critiques of capacity-building initiatives. For example, in the context of competency training in the linguistic field, Park [111] cautions on the potential colonization of competency-based trainings and encourages “fundamentally questioning competency-based trainings’ overwhelming emphasis on positivist analysis of linguistic knowledge and practice,” to decrease the risk of contributing to reproducing inequalities.

There are at least three related points about competency-based initiatives for the D&I fields to reflect on. First, not every researcher who does work that could be conceptualized as D&I self-identifies as D&I researcher [10, 112]. We do not want to exclude investigators in the fields simply because they do not have specific D&I competencies. In other words, we do not want to increase the silo that limits collaboration between D&I experts and other scholars. Second, the competencies identified in this study were created with Western, North American, and European perspectives, and as such, they may perpetuate colonizers’ theories and views [113]. As we continue to develop capacity-building initiatives, we need to pay attention to issues of power, marginalization, and oppression, and include reflexivity in the trainings [15, 105, 113–115]. We need to critically examine how we can collaboratively develop capacity building initiatives with community members, healthcare leaders, and policy makers [15] to decrease the gap between researcher and practice globally [32]. Third, competencies can be tied to “meritocracy,” which could have a negative effect on the development of the fields. Meritocracy places judgement on people’s knowledge, skills and ability to accomplish certain tasks. A challenge in such a system is identifying who has ownership of defining the ability of others, as this has consequences around hiring and retention in the workforce. The fields are in an early stage of developing competencies for different individuals working with the D&I science, and we hope that leaders can attend to the above cautionary points as they foster D&I capacity buildings.

### Recommendations

The fields of D&I have an incredible opportunity to make fundamental changes from within the science and the walls of academia to truly decrease the quality gap. Based on the data from this study, we propose that the D&I fields could benefit from:

1. clear consensus-based definition of competencies,
2. further development of capacity-building initiatives for various audiences, including researchers, practitioners, and operational leaders,
3. comparing and contrasting learner competencies for research and practice, and
4. critical thinking about how to embed equity in capacity building initiatives.

### Limitations

First, we did not conduct a systematic review and therefore may have missed important publications about capacity building in D&I, but this mapping review can inform the strengths and gaps in the fields. Second, the articles identified were in English, and therefore we could have missed other relevant literature. Third, we recognize that not all capacity-building initiatives in D&I are being published, or published in gray literature, and therefore the sample in this study is biased. Fourth, the search was conducted using only one database and could be strengthened by using a more systematic approach. Nonetheless, we have identified important points for discussion and enhancement in the D&I capacity building initiatives in our sample.

## Conclusion

Overall, we found that the competencies currently articulated in D&I capacity building initiatives infrequently differentiate by desired level of expertise, commonly focus on training researchers-only to the exclusion of practitioners, and infrequently and inconsistently incorporate critical areas such as equity and collaboration with other disciplines. As the fields continues to foster capacity-building programs, it will be important to think critically about the types of competencies we are developing for whom, how, and why.

## Data Availability

The datasets used and/or analyzed during the current study are available from the corresponding author on reasonable request.

## Abbreviation

D&I: Usually refers to dissemination and implementation, however here we are using it to refer to dissemination, implementation and translation
