# Correction: A mapping review and critique of the literature on translation, dissemination, and implementation capacity building initiatives for different audiences

**DOI:** 10.1186/s43058-025-00740-x

**Published:** 2025-04-25

**Authors:** Ana A. Baumann, Danielle R. Adams, Laura-Mae Baldwin, Rachel G. Tabak, Sara Malone, Maura M. Kepper, Anita D. Misra-Hebert, Kathleen R. Stevens, Maria E. Fernandez, Sunil Kripalani

**Affiliations:** 1 Division of Public Health Sciences, Department of Surgery, Washington University School of Medicine, St. Louis, MO USA; 2 School of Social Work, College of Health Sciences, University of Missouri, Columbia, MO USA; 3 Department of Family Medicine, University of Washington, Seattle, WA USA; 4 School of Public Health, Washington University in St. Louis, St. Louis, MO USA; 5 Cleveland Clinic Lerner College of Medicine of Case Western Reserve University School of Medicine, Cleveland, OH USA; 6 University of Texas Health Science Center San Antonio, San Antonio, TX USA; 7 Institute for Implementation Science, University of Texas Health Science Center at Houston, Houston, TX USA; 8 Vanderbilt University Medical Center, Nashville, TN USA

**Correction** **:** **Implement Sci Commun 6, 34 (2025)**

**https://doi.org/10.1186/s43058-025-00717-w**

After the original article was published, the authors reported that the name of author Maura M. Kepper had been misspelled as Maura M. Keeper. Furthermore, the details of Affiliation 4 have also been corrected, as it should read: School of Public Health, Washington University in St. Louis, St. Louis, MO, USA.

Authors Maura M. Kepper, Sara Malone and Rachel Tabak should be listed only as affiliated with Affiliation 4.

The original article [1] has been corrected.
